# A Framework for Automatic Morphological Feature Extraction and Analysis of Abdominal Organs in MRI Volumes

**DOI:** 10.1007/s10916-019-1474-3

**Published:** 2019-11-12

**Authors:** Hykoush Asaturyan, E. Louise Thomas, Jimmy D. Bell, Barbara Villarini

**Affiliations:** 1 School of Computer Science and Engineering, University of Westminster, London, UK; 2 School of Life Sciences, University of Westminster, London, UK

**Keywords:** 3D reconstruction, MRI, Automatic organ segmentation, Computer-aided diagnosis, Curvature, Volume

## Abstract

The accurate 3D reconstruction of organs from radiological scans is an essential tool in computer-aided diagnosis (CADx) and plays a critical role in clinical, biomedical and forensic science research. The structure and shape of the organ, combined with morphological measurements such as volume and curvature, can provide significant guidance towards establishing progression or severity of a condition, and thus support improved diagnosis and therapy planning. Furthermore, the classification and stratification of organ abnormalities aim to explore and investigate organ deformations following injury, trauma and illness. This paper presents a framework for automatic morphological feature extraction in computer-aided 3D organ reconstructions following organ segmentation in 3D radiological scans. Two different magnetic resonance imaging (MRI) datasets are evaluated. Using the MRI scans of 85 adult volunteers, the overall mean volume for the pancreas organ is 69.30 ± 32.50*cm*^3^, and the 3D global curvature is (35.23 ± 6.83) × 10^−3^. Another experiment evaluates the MRI scans of 30 volunteers, and achieves mean liver volume of 1547.48 ± 204.19*cm*^3^ and 3D global curvature (19.87 ± 3.62) × 10^− 3^. Both experiments highlight a negative correlation between 3D curvature and volume with a statistical difference (*p* < 0.0001). Such a tool can support the investigation into organ related conditions such as obesity, type 2 diabetes mellitus and liver disease.

## Introduction

Computer-aided diagnosis (CADx) or detection (CADe) systems are readily employed in life sciences, biomedicine and forensic science [1], to process medical image data for analytical purposes. For example, the computer-aided segmentation and classification of differences in organ morphology for patients with type 2 diabetes mellitus [2], polycystic liver disease [3], renal disease and obesity [4] has played a key role in supporting biomedical research. Moreover, recent literature reports that the measure of abnormal curvature growth can be used to stratify the severity and stages of Peyronie’s disease [5, 6]. The mean curvature of liver is used as an estimator for predicting the probability of suffering from and the severity of steatohepatitis [7]. Data about the structure and volume of psoas muscle can act as a predictor of outcomes for patients treated by chemotherapy for bladder cancer [8] and ovarian cancer [9].

Recent literature reports that the 3D brachial plexus reconstruction [10] determines the individual brachial plexus anatomy with maximum accuracy. Similar techniques are used to investigate craniocerebral trauma [11]. Furthermore, thin slice 3D reconstruction improves the detection of tumour margins in breast cancer patients compared to 2D CT images [12].

Considering the above successful applications of 3D rendering, the enhanced accuracy of 3D organ reconstruction in combination with morphological feature-based classification, may reveal previously unknown correlations between factors such as volume, curvature, spatial dimensions, anthropometry and health status. Although there are publications about varying correlations between organ-related morphological features and associated medical conditions, there are limitations to such studies, including a small patient dataset.

Recently, the relationship between nonalcoholic fatty liver disease (NAFLD) in type 2 diabetes has been investigated [13–15] together which increases the likelihood of developing complications of diabetes as well as augmenting the risk of more severe NAFLD [16]. Given the rising prevalence of type 2 diabetes [17, 18] and the broad clinical spectrum of the condition, there is a driving need for an improved understanding between morphology and function: this could provide objective measurements that help to establish progression, severity and remission.

### Contributions

In order to investigate the possible relationship between organ morphology and anthropometry, this paper proposes a framework for automatic morphological feature extraction in 3D organ reconstructions in 3D radiological scans.

Current research literature, to the best of our knowledge, has not reported a generalisable framework for morphological feature extraction and correlative analysis. Thus, the proposed framework expands on the methodology presented in [19] by integrating an automatic organ segmentation approach [20], and furthermore, employs multiple case studies to analyse the relationship between computed morphological features. Moreover, the proposed tool presented in this paper can easily integrate into medical image analysis systems, and provide a potential “second opinion” before a final diagnosis. The analysis of organ curvature could provide quantitative guidance towards the assessment and stratification of a medical condition, thus enabling an improved therapy plan.

This novel approach produces detailed boundary tracing (contouring) for every protrusion and indentation as opposed to an approximate rating of the organ, as verified by an independent senior radiologist and radiographer. Two diverse MRI datasets are evaluated for the pancreas and liver, demonstrating the effective generalisability of the proposed segmentation approach. Computing morphological features following 3D organ reconstruction highlight a negative correlation between 3D curvature and volume with a statistical difference (*p* < 0.0001). The implementation will be available at https://github.com/med-seg/morph-feat-extract.

Section “Methods” details the methodology for automatic organ segmentation, reconstruction and morphological feature computation. Section “Results and discussion” presents and discusses two sets of computations. Section “Conclusions” provides a conclusion for the proposed framework, including probable future work.

## Methods

**Fig. 1:**
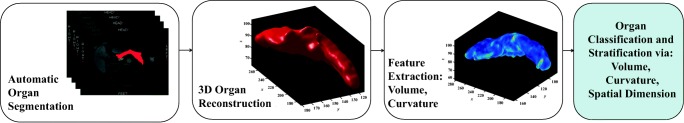
Overview of stages described in proposed framework. The organ is segmented, reconstructed and analysed to extract, classify and stratify morphological information

The proposed framework consists of four stages: (1) an organ of interest is automatically segmented in an MRI volume; (2) an accurate 3D reconstruction of the organ of interest is generated; (3) rich organ features relating to the shape, surface (texture) and dimension are extracted; (4) these extracted features represent key imaging biomarkers that can be utilised for enhanced classification and study of the organ [21, 22]. A schematic representation of the sequence of stages in the proposed framework is provided in Fig. 1.

Every stage in the framework can be treated as an independent process, potentially incorporated into another framework or pipeline. Replacing any stage in the framework will not significantly impact the complexity of a previous or later stage. The high modulatory enables a smooth substitution of different segmentation approaches without affecting the connections of subsequent stages. Moreover, the automatic segmentation stage is scalable depending on training data availability and capable of combining data augmentation. Both the 3D reconstruction and morphological-feature extraction stage can employ either manual or automatic segmentation using the target objects of interest.

### Automatic organ segmentation

The automatic organ segmentation methodology exploits a training dataset of expert-led manually annotated MRI volumes, that is, a dataset of contours for each organ of interest. The methodology of this technique [20, 23] progresses through three main stages. Firstly, a digital contrast is applied to a test MRI volume to enhance the organ tissue against background classes of non-organ tissue; furthermore, a bounding region is generated to identify (and isolate) the major organ area for every 2D slice in the test volume. Secondly, 3D image segmentation is performed via continuous max-flow and min-cuts approach [24, 25] to generate distinct segments of detailed boundary tracing. In order to retain organ tissue and eliminate maximum surrounding tissue, the boundaries or contours of segmented tissue are detected using structured forests [26–28]. Measures of similarity between segmented tissue contours and the manually annotated contours are computed using modified Hausdorff distance [29] and structural similarity index [30, 31] to yield a rough segmentation outcome as a volume. Thirdly, any remaining tissue deemed as non-organ of interest is eliminated via morphological operations including area, curvature and position between distinct tissue in the segmented organ volume.

### Reconstructing the organ

A 3D binary volume is generated from the segmented MRI volume. In attempt to reduce image noise, the reconstruction process employs a Gaussian smoothing algorithm that is applied to the 3D interpolated data. Using such a smoothing technique enhances image structures at varying scales of visualisation [32] as a pre-processing stage in computer vision. The equation of a 3D Gaussian function is

1 $$  G(x,y,z)=\frac{1}{2\pi\sigma^{2}}{\textstyle \exp\left( -\frac{x^{2}+y^{2}+z^{2}}{2\sigma^{2}}\right)} $$

where *x*, *y* and *z* are the distances from the origin in the horizontal axis, vertical axis and depth axis, respectively, and *σ* is the standard deviation of the Gaussian distribution. The values from the Gaussian distribution are used to build a convolution array that is applied to the original organ data. The Gaussian-based smoothed organ data is described as an isosurface by a set of points, for which the function represented by that data takes on a standard isovalue. In order to construct the isosurface, the marching cubes algorithm initially takes eight neighbour locations at a time, generates an imaginary cube and then determines the polygons needed to embody the part of the isosurface passing through this cube. Next, the individual polygons are fused into the desired surface by creating an index to an array of 256 possible polygon configurations in the imaginary cube. Each one of the eight scalar values represents 1-bit in an 8-bit integer: this bit is set to 1 if the value of the scalar is higher than the isovalue; otherwise, the bit is set to 0. Once all eight scalars have been analysed, the final value is the actual index to the polygon indices array. Each vertex of the created polygons is placed on the appropriate position along the edge of the imaginary cube by performing linear interpolation on the two scalar values connected by that edge.

Laplacian smoothing is then applied, producing the Laplacian of the rectangular 3D grid-based mesh. The implementation of a Laplacian algorithm returns an array of vertices coordinates: a new position is chosen for each vertex in the mesh using local information that relates to the position of neighbouring vertices. For each vertex, the smoothing operation is

2 $$  \overline{v}_{i}=\frac{1}{N}\sum\limits_{j=1}^{N}\overline{v}_{j} $$

where *N* is the number of adjacent vertices to node *i*, $\overline {v}_{j}$ is the position of the *j*-th adjacent vertex and $\overline {v}_{i}$ is the new position for node *i*. Once the 3D reconstruction process is complete, the 3D organ model can be visualised against a slice (2D image) from the original non-annotated MRI volume.

### Computing the volume and curvature

The total organ surface area of each slice is calculated as its respective segmented pixel area, and the organ volume per section is calculated as the product of each organ slice area and the MRI section thickness.

**Fig. 2:**
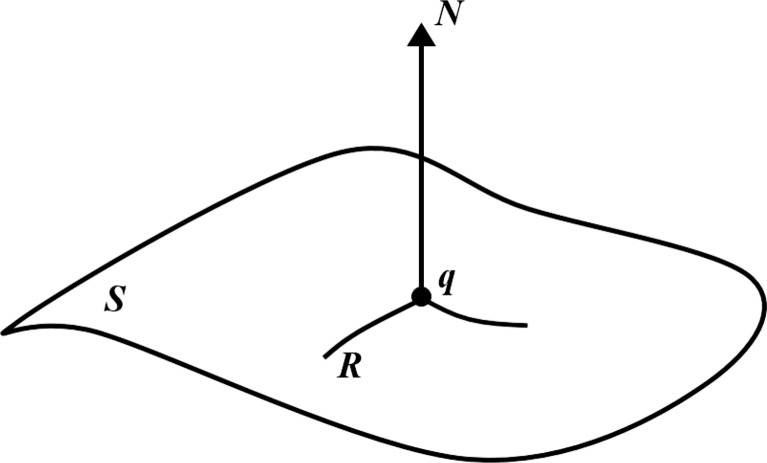
A point ***q*** is at a surface ***S*** with unit length normal ***N*** [33]. A regular curve ***R*** on the surface ***S*** passes through point ***q***

The 3D curvature of the organ is calculated on a triangular mesh [34], in which the curvature of a surface describes the local shape of that surface. A regular surface ***S*** is represented by ***d***(*x*,*y*) in Fig. 2, in which the point ***q*** lies on this surface. The orientation of ***S*** at ***q*** is the unit length normal, ***N***. Also, ***R*** describes a regular curve on ***S***, parameterised by *β*(*a*) = ***d***(*x*(*a*),*y*(*a*)) and where *a* is the arc length of ***R*** and *β*(0) = ***q***. Every curve that lies on the surface ***S*** at a given point of ***q*** ∈***S*** has the same tangent line and the same normal curvatures. Consequently, the normal curvature is referred to along a given direction at ***q***.

If ***q***_1_ with unit length surface normal ***N***_1_ is another different point on the surface very close to ***q***, and ***t*** is the normalised projection of the vector $\left (\boldsymbol {q}_{1}-\boldsymbol {q}\right )$ onto the tangent plane at ***q***, the normal curvature, *c*_*n*_(***t***), along the tangent direction ***t*** can be approximated as

3 $$  c_{n}(\boldsymbol{t})=-\frac{<\boldsymbol{q}_{1}-\boldsymbol{q}, \boldsymbol{N}_{1}-\boldsymbol{N}>}{\Vert\boldsymbol{q}_{1}-\boldsymbol{q}\Vert^{2}} $$

where,

4 $$  \boldsymbol{t}=\frac{\left( \boldsymbol{q}_{1}-\boldsymbol{q}\right)-<\boldsymbol{q}_{1}-\boldsymbol{q}, \boldsymbol{N}>\boldsymbol{N}}{\Vert\left( \boldsymbol{q}_{1}-\boldsymbol{q}\right)-<\boldsymbol{q}_{1}-\boldsymbol{q}, \boldsymbol{N}>\boldsymbol{N}\Vert} $$

A triangular mesh, ***M*** = (***P***,***C***), is employed in the next stage and viewed as an approximation of an unknown smooth surface. Consider ***P*** as representing a set of data points, and therefore, ***C*** can be described as the connection of ***P*** to construct edges and faces in ***M***. The motivation is to estimate the principal directions and curvatures at the vertices of ***M***. Firstly, the normal vectors at the vertices of the triangular mesh are estimated. Let the triangular face in ***M*** be *f*. Secondly, each of these faces *f*_*i*_ has a corresponding unit length normal vector $\boldsymbol {N}_{f_{i}}$, and the triangular mesh is positioned so that the normal vectors point to the same side of the surface. The normal, *N*, at vertex ***q*** of ***M*** is a weighted average normal to the triangular faces adjacent to ***q***, as

5 $$  N=\frac{{\sum}_{i=1}^{m}w_{i}\boldsymbol{N}_{f_{i}}}{\Vert{\sum}_{i=1}^{m}w_{i}\boldsymbol{N}_{f_{i}}\Vert} $$

where $\boldsymbol {N}_{f_{i}}$ are the unit length normal to the triangles in the “one-ring” neighbourhood of ***q*** and *w*_*i*_ is the weight, which is chosen based on the centre of the triangle face, *f*_*i*_. The number of points in a set of one-ring neighbour vertices of ***q*** is represented by *m*. For each neighbour ***q***_*i*_ of ***q***, ***t***_*i*_ can be defined as the unit length projection of the vector $\left (\boldsymbol {q}_{i}-\boldsymbol {q}\right )$ onto the tangent plane as

6 $$  \boldsymbol{t}_{i}=\frac{\left( \boldsymbol{q}_{i}-\boldsymbol{q}\right)-<\boldsymbol{q}_{i}-\boldsymbol{q}, \boldsymbol{N}>\boldsymbol{N}}{\Vert\left( \boldsymbol{q}_{i}-\boldsymbol{q}\right)-<\boldsymbol{q}_{i}-\boldsymbol{q}, \boldsymbol{N}>\boldsymbol{N}\Vert}\quad(i=1,\text{...}, m) $$

From here, it is possible to approximate the normal curvature *c*_*n*_(***t***_*i*_) as

7 $$  c_{n}(\boldsymbol{t}_{i})=-\frac{<\boldsymbol{q}_{i}-\boldsymbol{q}, \boldsymbol{N}_{i}-\boldsymbol{N}>}{<\boldsymbol{q}_{i}-\boldsymbol{q}, \boldsymbol{q}_{i}-\boldsymbol{q}>}\quad(i=1,\text{...},m) $$

Thus, the curvature of the organ is calculated on a triangular mesh that is based on local neighbourhood elements and vertices. The size of the neighbourhood can heavily affect results, and therefore increasing the neighbourhood size provides less sensitivity to noise [35], whereas a smaller neighbourhood size delivers better curvature estimates for less noisy data. From here, a global curvature *C*_*g*_ is computed as the mean value of all local curvatures, *j* = [1,...,*N*_*g*_], such that

8 $$  C_{g} = \frac{1}{N_{g}}\sum\limits_{j=1}^{N_{g}}\boldsymbol{c_{j}} $$

The value of *C*_*g*_ can be used as a guide to estimate the roughness and smoothness of the organ surface.

## Results and discussion

The proposed approach employs two datasets of T2-weighted (fat-suppressed) abdominal MRI image volumes, obtained using a Siemens Trio 3T scanner. The organ of interest, either the pancreas or liver, has been manually annotated by an expert-operator in every image volume. It is noted that the volunteers who underwent MRI scanning were aged over 18 and displayed early signs of type 2 diabetes. The pancreas dataset consists of 185 image volumes, split into 100/85 for training/test evaluation. Every image volume in the dataset consists of 80 slices with 2.0*mm* spacing, with each slice of spatial size 320 × 260 and 1.3128*mm* pixel interval in the axial and sagittal direction. The liver dataset consists of 50 image volumes, split into 20/30 for training and testing. Every image volume in the dataset consists of 370 slices with 3.0*mm* spacing, with each slice of spatial size 224 × 173 and 2.2321*mm* pixel interval in the axial and sagittal direction.

For the pancreas, the automatic segmentation method achieves a mean Dice Similarity coefficient (DSC) ± standard deviation (SD) of 81.14 ± 6.54*%*, and a mean Jaccard Index (JI) ± SD of 68.60 ± 7.68*%*. Analysing the liver segmentations produces a mean DSC ± SD of 92.17 ± 4.42*%*, and the mean JI ± SD is 85.73 ± 6.63*%*. The automatically segmented contours are elaborated to generate an image volume and an approximated surface using the techniques described in Sections “Introduction” and “Methods”. A number of 50 neighbouring points is chosen to calculate the curvature.

### Organ contouring

**Fig. 3:**
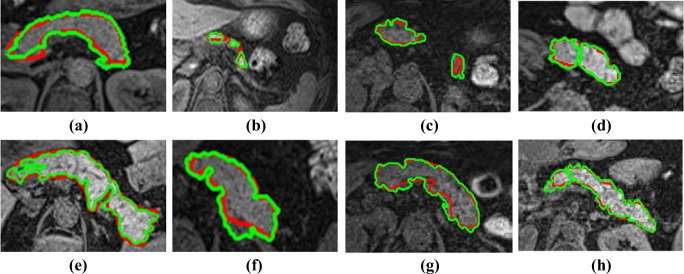
**a**, **b**, **c** and **d** display the segmentation (green) against ground-truth (red) contouring for eight slices in eight different pancreata, with a “smooth” surface. **e**, **f**, **g** and **h** the segmentation (green) against ground-truth (red) contouring for two slices in another two different pancreata, with a “ragged” surface

**Fig. 4:**
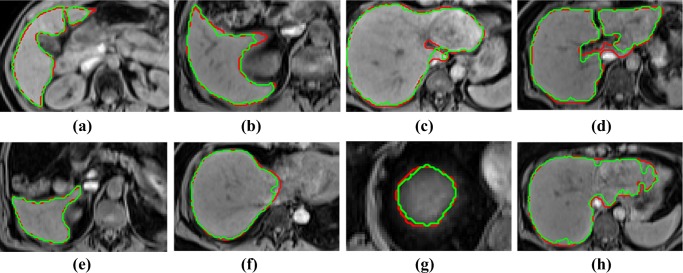
**a**–**h** display the segmentation (green) against ground-truth (red) contouring in eight slices from eight different livers

Figures 3 and 4 display the segmentation contouring (green) against the ground-truth (red) contouring in eight distinct slices in eight different pancreata and livers, respectively. The top row in Fig. 3 displays relatively smoother contouring in comparison to the bottom row. Furthermore, as verified by a senior radiologist and a senior radiographer, the segmentation output captures detailed protrusions and dents of the pancreas’ boundaries in comparison to the ground-truth.

Aiming to investigate and thus derive a possible organ classification model, the volume and 3D global curvature are calculated for each MRI scan (case). The extraction and potential classification of parameters, relating to shape and texture, can help to tackle fundamental clinical questions about organ changes associated with obesity and type 2 diabetes mellitus, which are coexisting conditions frequently associated with NAFLD disease. Furthermore, since NAFLD is an increasingly recognised condition that could progress to end-stage liver disease [36] improved image guidance systems could potentially help to assist final diagnosis.

**Fig. 5:**
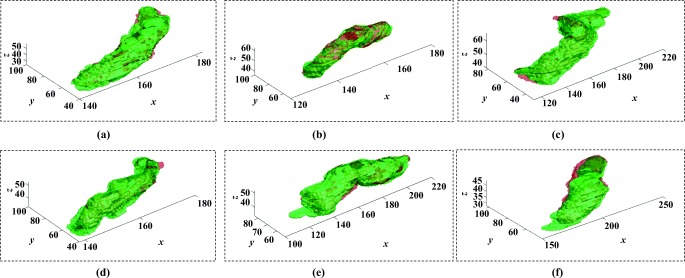
A list of six pancreas 3D reconstructions in six different MRI scans (volumes). The segmentation outcome (green) overlaps the ground-truth (red)

**Fig. 6:**
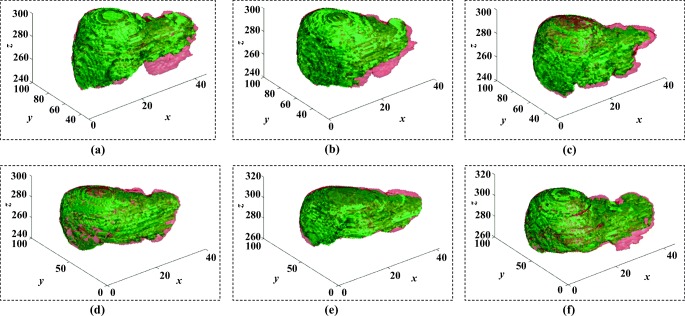
A list of six liver 3D reconstructions in six different MRI scans (volumes). The segmentation outcome (green) overlaps the ground-truth (red)

**Fig. 7:**
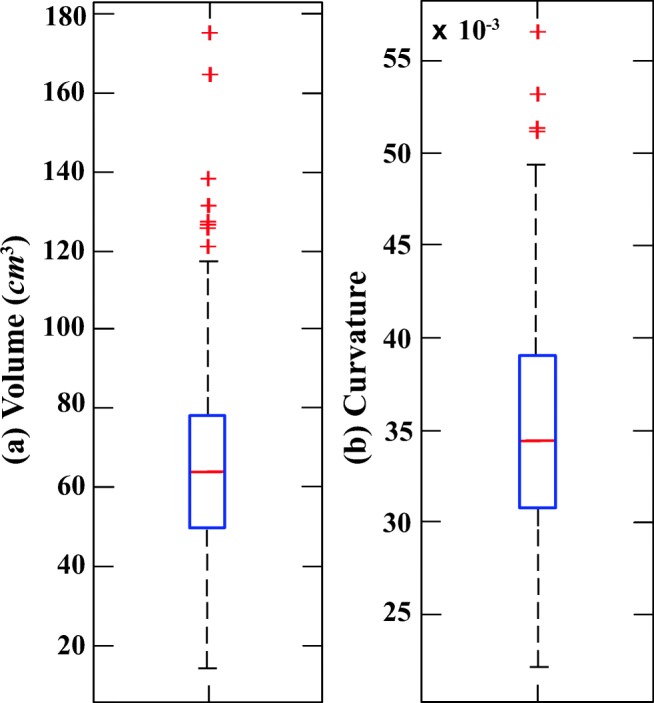
Box plots for (**a**) volume and (**b**) 3D curvature for 85 pancreas image volumes (cases)

**Fig. 8:**
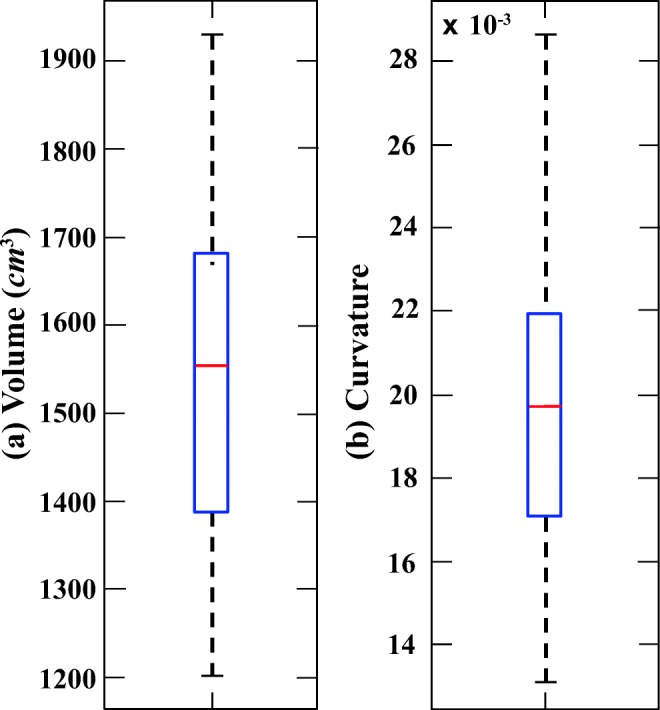
Box plots for (**a**) volume and (**b**) 3D curvature for 30 liver image volumes (cases)

Figures 5 and 6 illustrate six diverse volumetric pancreas and liver reconstructions, respectively, with the segmentation (green) overlapping the ground-truth (red). Notice the visibly high variation in pancreatic structure and curvature, which is also reflected in a higher curvature standard deviation (6.83 × 10^− 3^) compared to the standard curvature deviation for the liver dataset (3.62 × 10^− 3^) (Figs. 7 and 8).

### Analysis of automatic segmenation results

**Fig. 9:**
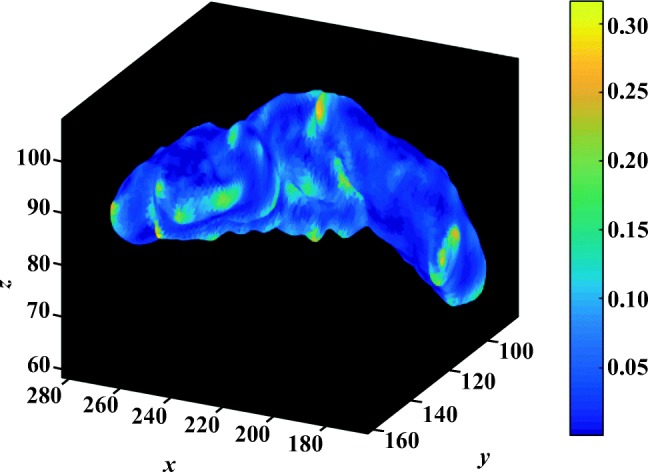
The depth of a pancreas curvature is highlighted. The varying shades of yellow indicate the highest degree of curvature dent (0.25 to 0.30) relative to global curvature of the organ

**Fig. 10:**
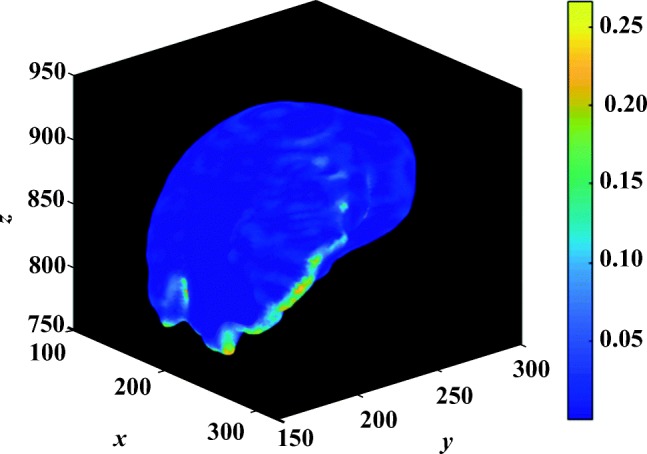
The depth of a liver curvature is highlighted. The varying shades of yellow indicate the highest degree of curvature dent (0.20 to 0.25) relative to global curvature of the organ

The following evaluation is based on automatic segmentation outcomes. The 3D reconstruction presented in Fig. 9 illustrates the depth of the pancreas curvature, with the “ragged” surface concentrated at the higher threshold with varying shades of yellow. Differences in pancreas volume may also be associated with age, gender and health status of the participants [37], and a wide variation in organ shape, size and curvature are established from this sample of 85 image volumes: the mean volume is 69.30 ± 32.50*cm*^3^ and the mean 3D global curvature is (35.23 ± 6.83) × 10^− 3^. Figure 7 provides a statistical representation of these computations. Interestingly, Fig. 10 illustrates relatively less variation in curvature compared to the curvature of the pancreas. Analysing the results from 30 image volumes achieves a mean volume of 1547.48 ± 204.19*cm*^3^ and a mean 3D global curvature of (19.87 ± 3.62) × 10^− 3^. A statistical representation of the data is provided in Fig. 8.

When comparing the automatic segmentation results to respective the ground-truth for evaluation purposes, Table 1 displays the MAE (mean absolute error), RMSE (root mean square error) and MAP (mean absolute percentage error) computations for volume and curvature in respect to corresponding ground-truth values of 85 pancreas and 30 liver image volumes.

**Table 1:** MAE (mean absolute error), RMSE (root mean square error) and MAP (mean absolute percentage error) computations for volume and curvature

Pancreas	MAE	RMSE	MAP (%)
Volume (*cm*^3^)	8.56	11.28	15.16 ± 13.17
Curvature (× 10^− 3^)	4.23	5.93	13.20 ± 13.46
Liver	MAE	RMSE	MAP (%)
Volume (*cm*^3^)	68.81	81.32	4.65 ± 2.94
Curvature (× 10^− 3^)	1.09	1.56	5.43 ± 4.83

### Analysis of morphological features

**Fig. 11:**
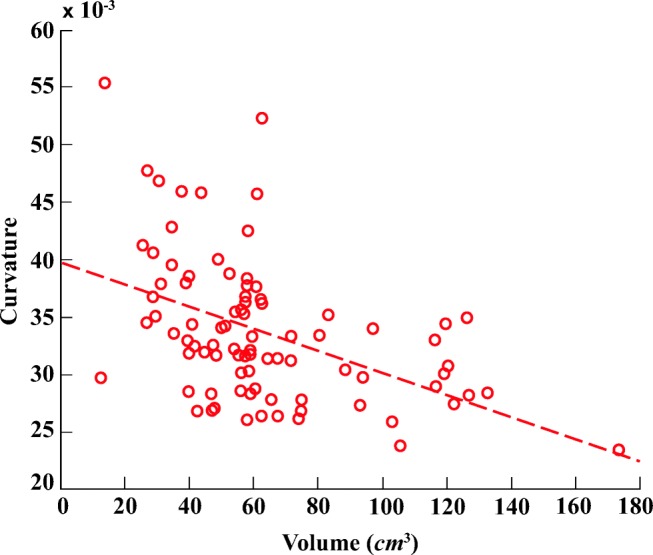
Ground-truth pancreas curvature in relation to volume a displays negative correlation

**Fig. 12:**
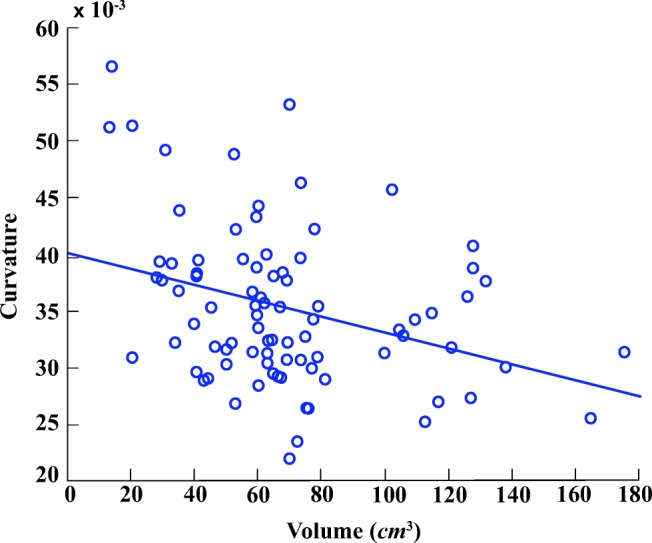
Automatic segmented pancreas curvature in relation to volume displays a negative correlation

**Fig. 13:**
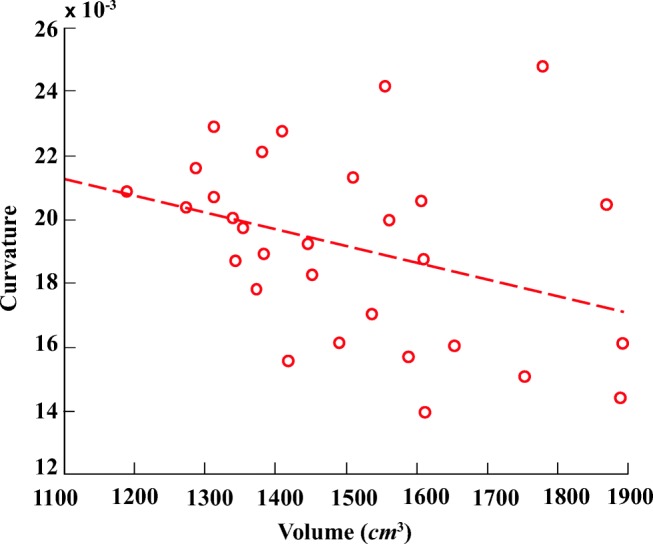
Ground-truth liver curvature in relation to volume displays a slight negative correlation

**Fig. 14:**
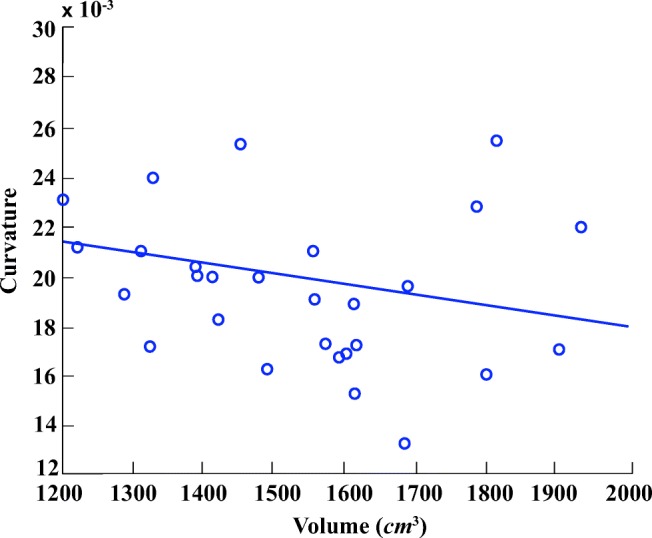
Automatic segmented liver curvature in relation to volume displays a slight negative correlation

In order to demonstrate the reliability of the proposed framework, the relationship between ground-truth morphological features is analysed alongside the morphology of automatic segmentation results. Figures 11 and 12 illustrate the relationship between pancreas volume and curvature for both the ground-truth (red) and the automatic segmentation (blue), respectively. As highlighted in both plots, the pancreas curvature versus volume displays a weak, but significant, negative correlation. Similarly, Figs. 13 and 14 illustrate a slight negative correlation for liver curvature and volume. For each dataset, the Wilcoxon signed-rank is computed using the differences between paired ground-truth volume and curvature values, and the differences between paired segmentation volume and curvature values. All four tests reveal a correlation that is statistically significant from zero (*p* < 0.0001).

Analysing the results presented in this paper, if the morphology of the pancreas can be described by the global curvature of the organ shape, then increasing values of global curvature are to be anticipated with decreasing organ size and thus giving rise to shape irregularities. Previous literature [38] reports that, for some patients with type 2 diabetes, there was an observed change in pancreas volume and morphology, in which the organ displayed an involuted morphology with serrated borders. Given the increase in global incidences of type 2 diabetes [17], there is a significant need to better understand the relationship between morphology and function. Furthermore, since NAFLD and type 2 diabetes mellitus frequently coexist and share the abnormalities of excess adiposity and insulin resistance, it can be necessary to compute objective measurements to stratify condition progression and remission, especially where changes in organ morphology have been observed but not quantified. Consequently, there is an apparent demand for objective methodologies that produce reliable morphological characterisations of the pancreas, liver and other major organs.

The segmentation program ran via a workstation with i7-59-30k-CPU at 3.50 GHz, and the mean time for segmenting the pancreas and liver is 25 and 32 minutes, respectively. Also, the computation of morphological measurements of volume and curvature is approximately 3 minutes. Using a GeForce Titan X GPU could potentially result in a tenfold decrease in run-time.

## Conclusions

Accurate 3D organ reconstruction, coupled with the analysis of size and structure, can support medical professionals perform clinical detection, diagnosis and planning of treatment. This paper describes a computing tool that automatically extracts and analyses organs based on morphological features using anonymised volunteer data from magnetic resonance imaging (MRI) volumes. In addition to offering radiologists a 3D organ reconstruction view and a “second opinion”, the proposed computational tool can easily integrate into forensic science and biomedical research. Although the case studies in this paper focus on MRI modality, the proposed framework is extendable to computer tomography (CT) and ultrasound (US). Due to differences in the imaging acquisition of MRI, CT and US, the automatic segmentation approach may differ, but since the framework is modular, it is also adaptable to other anatomical structures. Future work aims to utilise the proposed framework in a large cohort of abdominal image volumes, and, explore the potential correlations between corresponding anthropomorphic and environmental factors, and organ structure and shape.

## References

[1] Bornik A, Urschler M, Schmalstieg D, Bischof H, Krauskopf A, Schwark T, Scheurer E, Yen K (2018). Integrated computer-aided forensic case analysis, presentation, and documentation based on multimodal 3d data. Forens. Sci. Int..

[2] Burute N, Nisenbaum R, Jenkins DJ, Mirrahimi A, Anthwal S, Colak E, Kirpalani A (2014). Pancreas volume measurement in patients with Type 2 diabetes using magnetic resonance imaging-based planimetry. Pancreatology.

[3] Cnossen WR, Drenth JPH (2014). Polycystic liver disease: An overview of pathogenesis, clinical manifestations and management. Orphanet J. Rare Dis..

[4] Geraghty EM, Boone JM, McGahan JP, Jain K (2004). Normal organ volume assessment from abdominal CT. Abdom. Imaging.

[5] Ohebshalom M, Mulhall J, Guhring P, Parker M (2007). Measurement of penile curvature in peyronie’s disease patients: Comparison of three methods. J. Sex. Med..

[6] Su J, Lu A, Bryson C, Rosoff J, Honig S (2016). 125 initial peyronie’s disease questionnaire bother scores do not correlate with degree of penile curvature in patients with peyronie’s disease. J. Sex. Med..

[7] Gallego-Durán R, Cerro-Salido P, Gomez-Gonzalez E, Pareja MJ, Ampuero J, Carmen Rico M, Aznar R, Vilar-Gomez E, Bugianesi E, Crespo J (2016). Imaging biomarkers for steatohepatitis and fibrosis detection in non-alcoholic fatty liver disease. Sci. Rep..

[8] Zargar H, Almassi N, Kovac E, Ercole C, Remer E, Rini B, Stephenson A, Garcia JA, Grivas P (2017). Change in psoas muscle volume as a predictor of outcomes in patients treated with chemotherapy and radical cystectomy for muscle-invasive bladder cancer. Bladder Cancer.

[9] Yoshikawa T, Takano M, Miyamoto M, Yajima I, Shimizu Y, Aizawa Y, Suguchi Y, Moriiwa M, Aoyama T, Soyama H (2017). Psoas muscle volume as a predictor of peripheral neurotoxicity induced by primary chemotherapy in ovarian cancers. Cancer Chemotherap. Pharmacol..

[10] Van de Velde J, Bogaert S, Vandemaele P, Huysse W, Achten E, Leijnse J, De Neve W, Van Hoof T (2016). Brachial plexus 3D reconstruction from mri with dissection validation: a baseline study for clinical applications. Surg. Radiol. Anat..

[11] Li Z, Zou D, Zhang J, Shao Y, Huang P, Chen Y (2015). Use of 3D reconstruction of emergency and postoperative craniocerebral CT images to explore craniocerebral trauma mechanism. Forens. Science Int..

[12] Zhang Y, Zhou Y, Yang X, Tang P, Qiu Q, Liang Y, Jiang J (2013). Thin slice three dimentional (3D) reconstruction versus CT 3D reconstruction of human breast cancer. Indian J. Med. Res..

[13] Mantovani A, Byrne CD, Bonora E, Targher G (2018). Nonalcoholic fatty liver disease and risk of incident type 2 diabetes: a meta-analysis. Diabetes Care.

[14] Giorda C, Forlani G, Manti R, Mazzella N, De Cosmo S, Rossi MC, Nicolucci A, Russo G, Di Bartolo P, Ceriello A (2017). Occurrence over time and regression of nonalcoholic fatty liver disease in type 2 diabetes. Diabetes/metabolism Res. Rev..

[15] Noureddin M, Rinella ME (2015). Nonalcoholic fatty liver disease, diabetes, obesity, and hepatocellular carcinoma. Clin. Liver Disease.

[16] Hazlehurst JM, Woods C, Marjot T, Cobbold JF, Tomlinson JW (2016). Non-alcoholic fatty liver disease and diabetes. Metabolism.

[17] Bonini MG, Sargis RM (2018). Environmental toxicant exposures and type 2 diabetes mellitus: two interrelated public health problems on the rise. Curr. Opinion Toxicol..

[18] Candler TP, Mahmoud O, Lynn RM, Majbar AA, Barrett TG, Shield JPH (2018). Continuing rise of type 2 diabetes incidence in children and young people in the UK. Diabet. Med..

[19] Villarini, B., Asaturyan, H., Thomas, E. L., Mould, R., and Bell, J. D.: A framework for morphological feature extraction of organs from mr images for detection and classification of abnormalities. In: 2017 IEEE 30th International Symposium on Computer-Based Medical Systems (CBMS), pp. 666–671. IEEE, 2017.

[20] Asaturyan H, Gligorievski A, Villarini B (2019). Morphological and multi-level geometrical descriptor analysis in CT and MRI volumes for automatic pancreas segmentation. Comput. Med. Imaging Graph..

[21] Macauley M, Percival K, Thelwall PE, Hollingsworth KG, Taylor R (2015). Altered volume, morphology and composition of the pancreas in type 2 diabetes. PloS one.

[22] Neijenhuis MK, Kievit W, Verheesen SMH, D’Agnolo HM, Gevers TJG, Drenth JPH (2018). Impact of liver volume on polycystic liver disease-related symptoms and quality of life. United Europ. Gastroenterol. J..

[23] Asaturyan, H., and Villarini, B.: Hierarchical framework for automatic pancreas segmentation in MRI using continuous max-flow and min-cuts approach. In: International Conference Image Analysis and Recognition, pp. 562–570. Springer, 2018.

[24] Yuan, J., Bae, E., and Tai, X. -C.: A study on continuous max-flow and min-cut approaches. In: 2010 IEEE Computer Society Conference on Computer Vision and Pattern Recognition, pp. 2217–2224. IEEE, 2010.

[25] Yuan J, Schnörr C, Steidl G (2007). Simultaneous optical flow estimation and decomposition. SIAM J. Sci. Comput..

[26] Dollár, P., and Zitnick, C. L.: Structured forests for fast edge detection. In: Proceedings of the IEEE International Conference on Computer Vision, pp. 1841–1848. IEEE, 2013.

[27] Nair, B.: Fast edge detection using structured forests. International journal of Emerging Trends in Science and Technology, 2016

[28] Lawrence Zitnick C, Dollár P (2014). Edge Boxes: Locating Object Proposals from Edges.

[29] Dubuisson, M. -P., and Jain, A. K.: A modified hausdorff distance for object matching. In: Proceedings of 12th International Conference on Pattern Recognition, Vol. 1, pp. 566–568. IEEE, 1994.

[30] Dosselmann R, Yang XD (2011). A comprehensive assessment of the structural similarity index. SIViP.

[31] Chen M-J, Bovik AC (2010). Fast structural similarity index algorithm. J. Real-Time Image Proc..

[32] Wink AM, Roerdink JBTM (2004). Denoising functional MR images: A comparison of wavelet denoising and Gaussian smoothing. IEEE Trans. Med. Imag..

[33] Dong C-s, Wang G-z (2005). Curvatures estimation on triangular mesh. J. Zhejiang Univ.-Sci. A.

[34] Lorensen WE, Cline HE (1987). Marching cubes: A high resolution 3D surface construction algorithm. SIGGRAPH Comput. Graph..

[35] Rusinkiewicz, S.: Estimating curvatures and their derivatives on triangle meshes. In: Proceedings. 2nd International Symposium on 3D Data Processing, Visualization and Transmission, 2004. 3DPVT 2004, pp. 486–493. IEEE, 2004.

[36] Angulo P (2002). Nonalcoholic fatty liver disease. England J. Med..

[37] Caglar V, Kumral B, Uygur R, Alkoc OA, Ozen OA, Demirel H (2014). Study of volume, weight and size of normal pancreas, spleen and kidney in adults autopsies. Foren. Med. Anatom. Res..

[38] Macauley M, Percival K, Thelwall PE, Hollingsworth KG, Taylor R (2015). Altered volume, morphology and composition of the pancreas in type 2 diabetes. PLOS ONE.

